# Management of Mass-Casualty Incidents in Nepal: A Qualitative Case Study of Three District Hospitals in Nepal

**DOI:** 10.1017/S1049023X23006209

**Published:** 2023-10

**Authors:** Prinka Singh, Hamdi Lamine, Sujan Sapkota, Awsan Bahattab, Anneli Eriksson

**Affiliations:** 1. Department of Global Public Health, Karolinska Institutet, Solna, Stockholm, Sweden; 2. CRIMEDIM-Center for Research and Training in Disaster Medicine, Humanitarian Aid and Global Health, Università Del Piemonte Orientale, 28100 Novara, Italy; 3. Department for Sustainable Development and Ecological Transition, Università Del Piemonte Orientale, 13100 Vercelli, Italy; 4. HERD International, Sainbu Awas Cr-10 Marga, Bhaisepati, Lalitpur, Nepal

**Keywords:** disaster preparedness, hospital disaster response, mass-casualty incidents, mass-casualty management, Nepal

## Abstract

**Introduction:**

The frequency of disasters world-wide has significantly increased in recent years, leading to an increase in the number of mass-casualty incidents (MCIs). These MCIs can overwhelm health care systems, requiring hospitals to respond quickly and effectively, often with limited resources. While numerous studies have identified the challenges in managing MCIs and have emphasized the importance of hospital disaster preparedness, there is a research gap in the preparedness level and response capacities of district hospitals in Nepal.

**Study Objective:**

This study attempts to fill this gap by understanding the perception of hospital staff in managing MCIs in district hospitals of Nepal.

**Methods:**

A qualitative case study was conducted in three district hospitals in Nepal. Semi-structured interviews were conducted with the hospital personnel, using an interview guide. An inductive thematic analysis was carried out to understand their perception on the most recent MCI management.

**Results:**

Three themes emerged from the data analysis: enablers in MCI management, barriers in MCI management, and recommendations for the future. Use of multiple communication channels, mobilization of entire hospital teams, mobilization of police in crowd control, presence of disaster store, and pre-identified triage areas were the major enablers that facilitated successful MCI management. Nonetheless, the study also revealed challenges such as a lack of knowledge on MCI response among new staff, disruptions caused by media and visitors, and challenges in implementing triage.

**Conclusion:**

This study emphasized the importance of hospital disaster preparedness in managing MCIs and highlighted the significance of overcoming barriers and utilizing enablers for an efficient response. The findings of this study can provide the basis for the Ministry of Health and Population Nepal and district hospitals to plan initiatives for the effective management of MCIs in the future.

## Introduction

The frequency of recorded disaster events has risen substantially in recent years.^1^ Each year from 1970 through 2000, an average of 90-100 reports of medium- and large-scale disasters were recorded.^1^ However, from 2001 through 2020, this number increased to 350-500 reports every year.^1^ Additionally, the global yearly number of disasters is expected to increase by 40% by 2030.^1^ Disasters often creates a sharp rise in the demand for health care services due to a variety of reasons, including physical injuries or disease outbreaks.^2^ Thus, as a result of the increased number of disaster events, mass-casualty incidents (MCIs) have also become more common globally.^3,4^

According to the World Health Organization (WHO; Geneva, Switzerland), MCI refers to any situation in a hospital that results in a higher number of patients than available local resources can handle using standard procedures. It necessitates special emergency preparations as well as additional or specialized services.^5^

Most MCIs are complicated events that have the potential to overwhelm the health care systems, necessitating hospitals to respond quickly and effectively to provide care to a large number of patients.^6–8^ Such incidents may result from natural disasters like earthquakes, hurricanes, or floods, or from man-made disasters such as road traffic accidents or terrorist attacks.^8^ Besides these, epidemic such as the coronavirus disease 2019 (COVID-19) pandemic also cause a surge in hospitalizations, leading to a burden on health care systems, resulting into MCIs in hospitals.^9^

During MCIs, hospitals play an important role in providing emergency medical care to the injured.^10^ Yet during such events, hospitals are frequently confronted with the challenge of responding rapidly and effectively to a sudden surge of patients, especially with limited resources.^11^ Numerous studies have identified the challenges in managing MCIs and have emphasized the importance of preparedness, coordination, and communication in managing such situations. Implementation of response plans that define roles and responsibilities, and integrate multiple stakeholders into one response system, is recommended to overcome the challenges faced during MCIs such as management of uncertainties, communication gap, and collaboration with stakeholders.^3,12–15^

Nepal is one of the world’s top 20 disaster-prone countries, both natural and man-made, and is ranked fourth and eleventh in the world in terms of climate change and earthquake vulnerability, respectively.^16^ Both natural and man-made disasters have resulted in MCIs in Nepal and such events have underscored the need for effective MCI management in the health care settings.^17,18^ Previous studies conducted in Nepal have emphasized the critical role of disaster preparedness in the health care sector.^19–22^ The findings indicate that effective management of MCIs requires training and awareness among health care staff including intra- and inter-sectoral coordination, and the implementation of hospital incident command systems.^19–22^

A serious and growing concern in Nepal is MCIs, where natural disasters and road accidents can have devastating consequences. District hospitals are the major health service providers in Nepal and are typically the first point of contact in such emergencies, providing essential care to the injured and saving lives. Notwithstanding the vital role that district hospitals play in managing MCIs, there is a substantial research gap regarding the preparedness level and response capacity of these hospitals. To address this gap, this qualitative case study aims to understand the perception of hospital staff in the management of MCIs in district hospitals of Nepal.

The study could provide valuable insights into the existing disaster preparedness status of district hospitals in Nepal to manage MCIs. The findings can also aid in the development of strategies to enhance the disaster preparedness and response capacity of hospitals, ultimately saving more lives and reducing the health impact of these incidents.

## Methods

### Study Design and Setting

To provide an in-depth understanding of the management of MCIs and explore the perception of health care workers involved in managing those incidents in three district hospitals of Nepal, a qualitative case study was conducted.^23^ The hospitals were randomly coded as Hospital A, Hospital B, and Hospital C. Hospital A is a 15-bedded hospital, whereas Hospital B and Hospital C are 34 and 50 bedded hospitals, respectively.

### Case Description

Data were collected for the most recent MCI reported by the medical superintendent (head of the hospital) in each hospital. Table 1 presents a description of the most recent MCI scenario for each hospital.

**Table 1.** Recent MCI Scenario of Hospital

Hospital	Nature of MCI	Date	Number of Patients	Number of Deaths	Referred Cases
Hospital A	Bus Accident	January 31, 2022	17	None	1
Hospital B	Second Phase of COVID-19 Pandemic	Started Mid-2021	>300	34	Data Not Available
Hospital C	Bus Accident	Mid-December 2021 (approximate date)	80	None	11

Abbreviations: COVID-19, coronavirus disease 2019; MCI, mass-casualty incident.

### Data Collection Process

Medical superintendents from each hospital were contacted at first to obtain their permission for the study and an agreement for their staff to participate. Data were collected from August 2022 through October 2022. Semi-structured interviews were conducted with the medical superintendent and head of the emergency department of each hospital, using an interview guide to understand their perception in management of the most recent MCI. In two hospitals, the head of the emergency department was unavailable for an interview despite repeated follow-ups, so nurses in-charge were interviewed instead. These health personnel were selected based on their critical role in disaster response and their overall familiarity in MCI management.

The interviewees were contacted via email or phone call, and interview dates and times were scheduled. All interviews were conducted in the Nepali language and were audio recorded. While some interviews were conducted virtually using Zoom (Zoom Video Communications, Inc.; San Jose, California USA), others were conducted in-person in the participants’ offices at the respective hospital. This is because some participants expressed more comfort with an in-person interview. Virtual interviews were recorded on the Zoom platform, while in-person interviews were recorded using a voice-recording application on a cell phone. All interviews were facilitated by the principal investigator (PI), who is a Nepalese female with academic degree and professional experience in the related field. Only the respondent and PI were present at the interview, and notes were also taken by PI at the time.

### Data Analysis

All recorded interviews were transcribed in Nepali and then translated into English before analysis. An inductive thematic analysis was conducted. The transcripts were read several times for familiarization, and the participants’ words were used to identify codes. This process was repeated for all transcripts, and a codebook was developed. After one week, the whole process was repeated again to create a second codebook, which was then compared with the first one to develop a final codebook. Then, the transcripts were read again, and the final codes were identified for all interviews. The codes were then organized into categories, and themes were identified. All analysis was done manually by the PI.

### Ethical Considerations

Ethical approval was obtained from the Nepal Health Research Council (NHRC; Kathmandu, Nepal; reference number 4290). Additionally, permission letters were obtained from each hospital, and verbal consent was taken from each respondent prior to data collection. Participation was voluntary, and the right of participants to refuse or withdraw from the study at any point during or after data collection was respected. Each participant was assigned a unique code to protect their identity, and the study data did not contain any personally identifying information.

## Results

### Demographic Information of Participants

Six participants from three different hospitals were interviewed. All participants were coded randomly as P1, P2, P3, P4, P5, and P6. The average time for the interview was 41 minutes. Table 2 summarized the demographic information of respondents. Most participants were doctors with 1-10 years of experience working in their respective hospitals. Most respondents managed 1-10 MCIs in their hospital. One-half of the participants had no prior training in disaster preparedness, whereas the other-half had training in trauma and mass-casualty management (MCM) planning.

**Table 2.** Demographic Characteristics of Participants

Respondent	Hospital	Academic Background	Years of Experience in Respective Hospital	Number of MCIs Managed in Respective Hospital	Past Disaster Training
P1	Hospital A	Doctor	1-10 years	1-10 incidents	None
P2	Hospital B	Paramedics	>10 years	>10 incidents	Trauma and MCM Training
P3	Hospital C	Nurse	>10 years	>10 incidents	MCM Training
P4	Hospital C	Doctor	1-10 years	1-10 incidents	None
P5	Hospital B	Doctor	1-10 years	1-10 incidents	MCM Training
P6	Hospital A	Doctor	1-10 years	1-10 incidents	None

Abbreviations: MCI, mass-casualty incident; MCM, mass-casualty management.

### Themes

Three themes emerged during data analysis: enablers in MCI management, barriers in MCI management, and recommendations for effective management of MCI in the future.

### Theme 1: Enablers in MCI Management

*Enablers in Communication—*For internal communication, most participants mentioned using social media platforms such as WhatsApp (Meta Platforms, Inc.; Menlo Park, California USA), Facebook (Meta Platforms, Inc.) groups, and Viber (Rakuten Group, Inc.; Tokyo, Japan) for quick information exchange regarding the MCI. In addition, a phone call to all departments in charge, verbal communication, and miking (use of a microphone to amplify the sound) were also used.

For external communication, most participants organized stakeholder meetings:

We did a meeting, and discussion with all journalists, deputy CDO [meaning Chief District Officer], and other local stakeholders. How was the situation, how many casualties were there, how was their condition, we informed everyone. *P4 added.*

A few participants also mentioned that they posted the information on the hospital’s noticeboard and Facebook page so that the public, journalists, and others were kept informed about the situation.

*Enablers in Human Resources—*All participants mentioned the mobilization of all staff as a key enabler:

We called all staff, ward nurses, off-duty nurses, we called everyone. There was of course staff inadequacy, but because of support from all staff, because of our good teamwork, we were able to manage that accident. *P6 explained.*

P1 and P6 noted that their staff were either the local people living near the hospital or were living inside the hospital accommodation, which made their mobilization easier during the MCI management.

Some participants also cited the designation of roles to staff as key facilitators:

To avoid confusion while managing the MCI, I assigned specific roles and responsibilities to each staff member. For instance, who is responsible for communication, who is responsible for managing the lab, for overseeing blood management, or ensuring proper medicine management. *Clarified P1.*

*Enablers in Safety and Security—*Most participants spoke on the need for the mobilization of police:

We also have a hospital police who coordinated with other police forces, and then an additional police team was mobilized inside the hospital. They managed the crowd very well. *Remarked P6.*

Public support was mentioned by a few participants:

Local volunteers promptly arrived to support us during the time of the accident. They helped us manage the crowd. *P3 elaborated.*

A few participants also mentioned using a one-way entry system and barricade tape for crowd control.

*Enablers in Logistics and Supply Chain Management—*The presence of disaster stores (storage area dedicated for the stockpiling of commodities to be used in the event of a disaster) was emphasized by all participants:

We follow MSS, a minimum service standard that mandatorily requires a disaster store to be stocked with the necessary medications and equipment. We managed accordingly, that’s why we were able to use those commodities. *Stated P3.*

For P5, external support from stakeholders and the public was an additional enabler:

Some individuals voluntarily came forward and donated to fill the oxygen cylinders, while others covered the transportation costs. Despite our hospital’s limited budget, we were able to manage with the generous help and support of the public. *P5 added.*

*Enablers in Triage and Surge Capacity—*Within triage and surge capacity, two enablers were identified. The first was the hospital infrastructures and facilities itself, where all participants cited that they had a pre-identified triage zone in their hospital. A few participants mentioned that they expanded their triage area to increase the surge capacity:

Our green area was OPD [meaning out-patient department], which was insufficient later on. So, we expanded the area by opening the OPD door and utilizing the doctor’s examination area. We put mattress there as well. *P3 expressed.*

The second enabler was the knowledge and abilities of the staff, which had a significant impact on proper patient care and management:

One patient in the green area was on the verge of going into shock, possibly due to fear. But our staff effectively managed that situation. I felt proud of their efforts. They ensured that the patient received timely care. They could have missed that patient in the chaos. *Claimed P3.*

*Enablers in Continuity of Essential Services—*Most participants expressed that they designated some staff for the continuity of essential services:

There will be of course on-going ER [meaning emergency room] treatment during the MCIs and such patients are often neglected at that time. We kept this thing in the back of our mind. That’s why, we designated one medical officer to manage the existing ER cases. *P4 noted.*

Other enablers were postponing the OPD services and providing essential services in parallel either by shifting the existing ER cases in the ward or by discharging them based on their condition.

*Other Enablers—*P3 and P4 stated how the organization of the drill in their hospital supported during the actual MCI management:

During the drill, we practiced patient triage in the red, yellow, and green zone. Two weeks after the drill, we encountered this bus accident, and our recent practice proved valuable in managing the situation effectively. *P4 added.*

### Theme 2: Barriers in MCI Management

*Barriers in Human Resources—*All participants identified staff inadequacy and overwork as major barriers in human resources. Another barrier was the newly recruited staff who were neither aware of the existing hospital disaster plan nor their roles and responsibilities for MCI management:

The new staff were inexperienced, and they had not received any training or orientation on our hospital disaster plan. That’s why they could not follow the instruction properly while managing the victims. They even require our [meaning trained and experienced hospital staff] frequent guidance on patient care. *Reported P6.*

*Barriers in Safety and Security—*Media management was one barrier:

While we were treating patients, some journalists took pictures without permission. When we asked them to leave, they became angry and even shouted at our doctors. *Expressed P4.*

Moreover, participants also encountered problems due to patient visitors:

They [meaning visitors] verbally abused us, demanding that we prioritize the care of their own patients first. That’s why some people were angry. *P6 stated.*

*Barriers in Logistics and Supply Chain Management—*Most participants stated the absence of a blood bank and computed tomography (CT) scan within the hospital resulted in patients being referred elsewhere, leading to delay in their treatment. Other challenges were delayed procurement of commodities, lack of electricity backup, and no replacement of commodities in the disaster store.

*Barriers in Triage and Surge Capacity—*Only P4 mentioned the barriers in triage and surge capacity. First, their hospital did not have plans for increasing surge capacity, and second, they could not perform triage for all victims:

While there was a team of doctors stationed outside the ER for triage, they were bypassed by the volunteers and police, who took the patients directly to the red zone thinking that the condition of the patient was serious. *P4 explained.*

*Other Barriers—*One significant barrier was the absence of municipal support. Concern was expressed by stating:

Municipality did not take any action to help us. Despite our requests for staff assistance, they did not provide any. They even did not visit us to understand how the situation is*. P2 expressed.*

For P2 and P5, lack of public support was a barrier:

The COVID-19 ward was situated near the community, and rumors began to circulate that the hospital was spreading the virus. Even the ward chairperson and some health staff members expressed these concerns, which was quite demotivating for us. *Stated P5.*

The transportation management committee (a local committee that is responsible for developing and implementing policies related to bus routes, schedules, fares, safety, maintenance, and other aspects of bus transportation) turned out to be another obstacle for P3 and P4:

Our hospital had limited funds for such incidents [meaning bus accident], and we asked the transportation management committee to bear the expenses. They initially refused to cover the expenses, which amounted to around 350 thousand rupees. Our hospital had to cover the expenses. This caused additional financial strain, as we were unable to replenish the disaster store’s inventory. Eventually, it took six months for us to receive payment from the committee. *P4 cited.*

### Theme 3: Recommendation for Effective Management of MCI in the Future

*Recommendation for Hospital—*All participants felt the need to organize a mock drill at a regular interval of time. Additional suggestions included involving ambulance drivers in the hospital disaster plan, fast coordination and communication with stakeholders, quick procurement of commodities, ensuring ample supplies of logistics in the disaster store, and establishing and maintaining a blood bank and CT scan within the hospital.

*Recommendation for Staff—*All participants emphasized the importance to regularly update and communicate regarding the hospital disaster plan to ensure that staffs are well-informed and prepared for an emergency:

Merely having one person follow the hospital disaster management protocol does not guarantee that others will do the same. Therefore, it is necessary to orient every staff member on the existing disaster plan to manage MCIs effectively. *Commented P1.*

*Recommendation for Stakeholders—*All participants recommended central government to provide more human resources to their hospitals:

We received 17 victims during that accident. So, we could manage it. But, if there had been a higher number of casualties, it would have been extremely challenging because neither we have sufficient manpower for it, nor did we have a nearby hospital for a referral. *Explained P6.*

Some participants also suggested the municipality to provide good support.

P5 suggested that the central level authorities should do a proper need assessment at ground level:

Amidst the COVID-19 pandemic, what we required was additional manpower, but instead, the central level sent us equipment. However, it wasn’t just the equipment that we needed, as we also required skilled personnel to operate it. Unfortunately, this kind of analysis seemed to be lacking in their [meaning central level authorities] decision-making process. *P5 expressed.*

## Discussion

The present study detailed the management of the most recent MCIs that occurred in three district hospitals in Nepal. The study identified several enablers in MCI management, such as using multiple communication channels for internal and external communication, mobilizing the entire hospital team for MCI response, involving the police in crowd control, maintaining a disaster store for logistic supplies, having a pre-identified triage area for patient prioritization, and managing existing ER cases parallelly for the continuity of essential services. However, the study also uncovered some significant barriers, such as newly recruited staff lacking knowledge of MCI response, disruption of work due to media and visitors, absence of a blood bank and CT scan in the hospital, no plan for expanding surge capacity, and challenges in implementing triage.

Disaster management relies on a collaborative team approach with clear roles and responsibilities assigned to each staff member involved in MCIs management.^24^ Usually during MCIs, hospitals mobilize their entire staff,^25,26^ an act that fosters a sense of teamwork and collaboration needed for effective MCI management, which is also the case in this study. However, staff inadequacy, excessive workloads, and the lack of knowledge and experience among newly recruited staff on the hospital’s disaster plan and their roles and responsibilities during MCIs are all challenges which small district hospitals frequently encountered. This causes confusion in the management process, hinders effective and efficient teamwork, and can result in suboptimal patient care.^27^ Therefore, it is essential to ensure that every staff member fully understands their roles and responsibilities during a disaster.^12^ For this, frequent staff training and orientation is necessary to ensure all staff members have the requisite skills and knowledge to manage the MCIs.^24,25,28^ In addition, it is also crucial to organize a regular and structured drill to prepare staff and ensure they are sufficiently trained and confident to respond to MCIs.^24,27,29^

Apart from internal teamwork, MCIs also require a collaboration and coordination with external stakeholders.^14^ The external support mentioned in this study from stakeholders and the general public, such as donations and covering transportation costs to fill up the oxygen cylinder during the COVID-19 pandemic, can be critical in situations where health care facilities have limited budgets. Such support can help bridge the gap and guarantee that the essential resources are available to effectively manage the disaster.^12^ As a result, hospitals must maintain a strong network of external stakeholders, such as local government, donors, or nongovernmental organizations, to provide assistance during emergencies.^14^

Moreover, to facilitate the internal and external teamwork, effective communication is equally essential.^14^ This study revealed that the use of multiple communication approaches, including social media, during MCIs facilitates quick information exchange and better coordination among hospital staff and stakeholders.^3,30^ Therefore, hospitals should consider incorporating both traditional and digital approaches into their communication strategies.

One barrier encountered during MCIs management in this study was unregulated media and patients’ visitors, with whom hospitals are often crowded at the time of MCIs. Despite the essential role of media during disasters in providing a timely and accurate information to the public, their presence may cause disruptions within the ER, as mentioned in the current study.^31,32^ Likewise, visitor presence can also create additional stress and disrupt the workflow of health care providers.^3^ In such scenarios, safety and security measures such as mobilization of police, use of barricade tape, and establishment of a one-way entry system should be considered.^3,30^ Apart from that, a separate area must be designated for media personnel within the hospital, preferably away from the ER.^13,31^ Additionally, hospitals can develop and implement protocols that clearly outline the role of the media during MCIs, including policies on photo and video recording, media access, and patient consent.^32^ Hospitals should also develop and implement visitor policies that include the restrictions on the number of visitors, visiting hours, and access to patient care areas.

Another challenge was the resource management. During MCIs, hospitals may need to reallocate their resources to deal with emergencies.^3^ Postponing OPD services can help free up resources that can be used to manage MCI.^3^ In addition, hospitals may need to transfer existing ER cases to the ward or discharge them, depending on their condition, to make a room for the incoming MCI cases.^24^ Also, hospitals’ adherence to minimum service standards, such as availability of disaster store stocked with necessary medications and equipment, ensures that hospitals are well-prepared for emergencies and have the necessary resources in hand.^3,30^ However, hospitals in the present study cited the insufficient human resources and logistics. This is concerning as it suggests that these hospitals would struggle to manage an increased number of casualties during the MCI. To address this issue, hospitals, in coordination with stakeholders, should prioritize the development of surge capacity plans that allow for the rapid expansion of resources and staffing to effectively manage a large number of patients during MCIs.^12^ Furthermore, despite having a pre-determined triage zone, triage procedures may be challenging to implement at the ER entrance when multiple victims appear at once because volunteers and police often bypass the triage team and enter the red area directly.^3,11^ This bypassing could result in some patients being placed in the wrong zone or not receiving the appropriate level of care.^3^ This could further strain the hospital’s resources and can negatively impact patient outcomes.^15^ Hence, it is important for hospitals to triage all victims to ensure that resources are allocated efficiently and that patients receive timely and appropriate care.^29^

## Strengths and Limitations

As far as the authors know, no study has been undertaken in Nepal to evaluate how the district hospitals have managed the MCIs. So, this study can contribute to the literature for future hospital disaster planning and preparedness initiatives in Nepal. Next, rather than relying on mock drills or tabletop exercises, a real MCI scenario that occurred within the hospital was considered to understand how district hospitals managed MCIs. Compared to simulated drills or tabletop exercises, a real-life MCI provides a more comprehensive understanding of the emergency response.

Despite these strengths, the study has several limitations. First, in spite of collecting data on the most recent MCI, recall bias cannot be entirely eliminated. Secondly, due to time constraints, only two staff members were interviewed from each hospital, which may have impacted the study’s findings, as involving all members of the hospital disaster management committee could have provided a more comprehensive picture of MCI management. Nevertheless, data saturation was reached, minimizing the bias. Lastly, the analysis was conducted by a single researcher, and although the coding process was repeated several times to minimize bias, having a sole coder may still have influenced the study outcome.

## Conclusion

Effective MCI management necessitates a coordinated and well-planned response that leverages enablers and overcomes barriers. This study offers useful insights into the factors that facilitate and impact the management of MCIs and emphasized the significance of education, awareness, and training for hospital disaster preparedness.
